# Hybrid Dilated Convolution with Multi-Scale Residual Fusion Network for Hyperspectral Image Classification

**DOI:** 10.3390/mi12050545

**Published:** 2021-05-10

**Authors:** Chenming Li, Zelin Qiu, Xueying Cao, Zhonghao Chen, Hongmin Gao, Zaijun Hua

**Affiliations:** College of Computer and Information, Hohai University, Nanjing 211100, China; lcm@hhu.edu.cn (C.L.); qiuzelin@hhu.edu.cn (Z.Q.); shary@hhu.edu.cn (X.C.); chenzhonghao@hhu.edu.cn (Z.C.); huazaijun@hhu.edu.cn (Z.H.)

**Keywords:** HSI classification, local and hybrid dilated convolution, residual fusion networks

## Abstract

The convolutional neural network (CNN) has been proven to have better performance in hyperspectral image (HSI) classification than traditional methods. Traditional CNN on hyperspectral image classification is used to pay more attention to spectral features and ignore spatial information. In this paper, a new HSI model called local and hybrid dilated convolution fusion network (LDFN) was proposed, which fuses the local information of details and rich spatial features by expanding the perception field. The details of our local and hybrid dilated convolution fusion network methods are as follows. First, many operations are selected, such as standard convolution, average pooling, dropout and batch normalization. Then, fusion operations of local and hybrid dilated convolution are included to extract rich spatial-spectral information. Last, different convolution layers are gathered into residual fusion networks and finally input into the softmax layer to classify. Three widely hyperspectral datasets (i.e., Salinas, Pavia University and Indian Pines) have been used in the experiments, which show that LDFN outperforms state-of-art classifiers.

## 1. Introduction

The technology of hyperspectral remote sensing makes full use of high-altitude detection equipment with visible light, infrared light and microwave and other technical methods through information processing and transmission, which can carry out the remote non-contact classification and recognition of ground objects. Hyperspectral image (HSI) has hundreds of adjacent narrow bands [1] that have a large number of channel dimensions, so it plays a significant role in the field of remote sensing. Hyperspectral image has important information on two sides: one is spectral information, which can provide the ability to differentiate land-cover materials, the other is spatial information which can provide rich information about the spatial structure. Therefore, HSI is applied widely in many domains, such as military exploration [2,3], agriculture [4,5], environment monitoring [6,7] and medical treatment [8].

In the early age of hyperspectral image classification, traditional machine learning methods were widely used, for example, support vector machines (SVM) [9,10], k-nearest neighbor (KNN) [11,12], multinomial logistic regression (MLR) [13,14], decision tree [15,16]. However, within the same material exist spectral differences in different spaces and different materials may have similar spectral characteristics, so the obtained maps are still noisy due to the limited ability of spatial structure feature extraction. In order to resolve the problem where it is difficult to effectively classify hyperspectral images only by spectral features, many methods of artificial extraction of spatial and spectral features are proposed, for example, Markov random fields (MRFs) [17], generalized composite kernel machine [18].

In recent years, with the development of technology, deep learning methods can provide more dynamic automation features. The basic idea of deep learning is that the training model resolves which features are more significant than others in the case of fewer human constraints. Therefore, deep learning methods have been widely used in HSI classification, for instance, M. He et al. [19] proposed a neural network that has a multi-scale 3D deep convolution for HSI classification that can learn 2D multi-scale spatial features and 1D spectral features from the HSI data end-to-end. S. Mei et al. [20] proposed an unsupervised 3D convolutional auto-encoder (3D-CAE) that designed a 3D decoder to reconstruct the input patterns and all parameters could be trained without marking training samples. In [21], a spectral-spatial residual network (SSRN) was put forward, which uses the original 3D cube as input data and continuously studies the distinguishing feature information of HSI through the spectral-spatial residual block. In [22], a contextual deep CNN (D-CNN) optimizes local context interaction by exploiting local spatial-spectral relationships of neighboring individual pixel vectors. In [23], a novel Synergistic CNN which fuses 2D and 3D networks was proposed for accurate HSI classification. In [24], a 3D CNN based on residual group channel and attention network was proposed for HSI classification, which strengthens the spatial features by extracting spatial context information and can reduce the loss of meaningful and useful information. 

These models use different methods on the basis of deep learning. However, with the depth of network layers becoming deeper, they face difficulties with training and accuracy decline.

Considering the problems above, the paper proposed a multi-scale feature fusion network based on local and hybrid dilated convolution (LDFN), which uses a fusion strategy that not only picks up the local information of details but also collects the rich spatial features by expanding the perception field. A residual fusion network was designed to integrate the local convolution and hybrid dilated convolution (HDC), which have a deeper structure of networks and fast connection with other layers. Therefore, our methods have great robustness and excellent ability to learn spatial-spectral information classification.

In summary, the main contributions of this paper are three-fold.

(1) The proposed hybrid dilated convolution stacked with different sizes of dilation rates is used to extract the spatial information.
(2) Local and hybrid dilated convolution methods are integrated which can simply replace the traditional standard convolution. Local convolution connects local pixels closely, which can make our convolution layer more flexible and expressive. Then hybrid dilated convolution is able to raise the field of vision without raising the amount of computation, which can fully collect the spatial-spectral features of hyperspectral images.
(3) The proposed new model also uses the specific residual network [25] to fuse the previous HDC and standard convolution on main channels, which can extract multi-scale fusion features.

The rest of this paper is arranged as follows. Section 2 discusses the related CNN methods and introduce the LDFN framework for HSI. Section 3 shows experiments over four benchmark hyperspectral datasets. Finally, conclusions are presented in Section 4.

## 2. Materials and Methods

### 2.1. Proposed Methods

Traditional CNN consists of several normal operations, such as convolution operations, activation operations, batch normalization operations and pooling operations. The details of the convolution operations are as follows.

(1) Convolutional Layers

The convolution layer is the most significant part of the convolution neural network. The input of each node is only a small part of the upper layer. Convolution layers analyze feature maps of the previous layer through the filters deeply and obtain more abstract features. Therefore, it can deepen the depth of the network. Let x be the input of data, and the size of input data is h×l×c, where h and l mean the height and width of the spatial feature, c represents the numbers of spectral channels. Let w and b represent the weight parameter and bias parameter. $y_{i}$ represents the oth layer output and *k* means kernel. The formula of the convolutional layer [26] is:

(1) $$y_{o}=\sum_{i=1}^{c}\sigma (x_{i}*w_{o}+b_{o}),o=1,2,\ldots k$$

It should be noted that σ represents the activation function.

(2) Dilated Convolution and HDC

While considering the classification algorithm, spatial-spectral characteristics should also be considered. In order to pick up hyperspectral features, standard convolution pays more attention to repetitive operations, which tremendously improves the computational complexity, and local convolution ignores the spatial similarity of adjacent regions.

**Figure 1:**
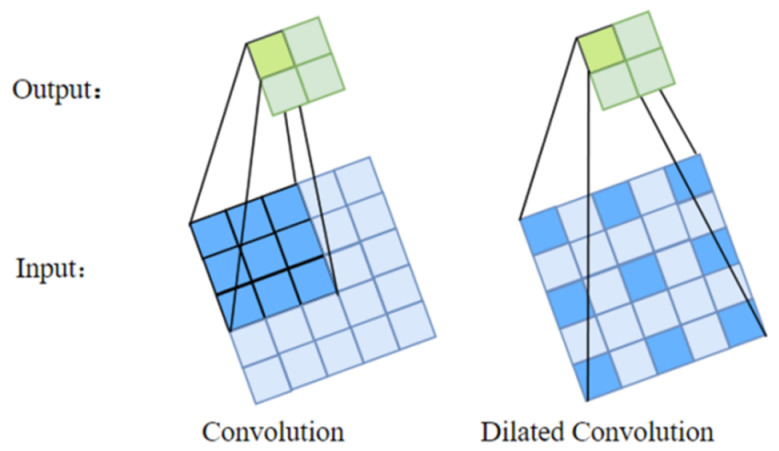
Standard and dilated convolution.

The comparison of standard and dilated convolution is revealed in Figure 1. Dilated convolution is able to expand the perception field of the convolution domain and capture multiscale context information, which is able to effectively settle the matter of insufficient spatial information extraction. However, traditional dilated convolution may lead to two problems, one is the gridding effect which means the kernel is not continuous, the other is that long-ranged information might be not relevant, this means that the method may be invalid on small objects. Therefore, a hybrid dilated convolution, which consists of different sizes of dilation rates, is proposed.

**Figure 2:**
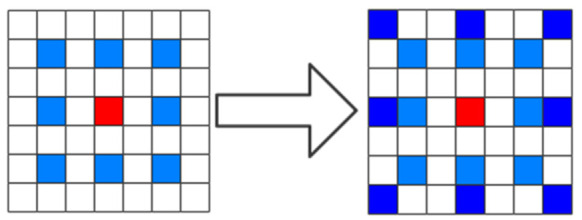
Hybrid dilated convolution.

Hybrid dilated convolution consists of different dilation rates that can effectively solve the problems above. Figure 2 illustrates the process from dilated convolution to HDC. HDC has three characteristics, first, the shape of the dilation rate is designed as a zigzag structure. Second, the dilation rates of stacked HDC cannot have a common measure of more than 1. The last, HDC satisfies a formula [27]:

(2) $$M_{i}=\max[M_{i+1}-2r_{i},M_{i+1}-2(M_{i+1}-r_{i}),r_{i}]$$

where $r_{i}$ represents the dilation rate of the ith layer. $M_{i}$ represents the largest dilation rate of the *i*th layer. With the integration of local convolution and HDC, full spatial information can be covered.

### 2.2. HSI Classification Based on LDFN

**Figure 3:**
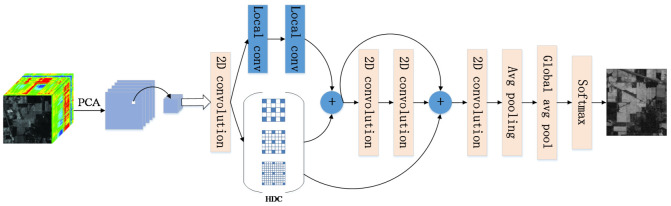
The flowchart of the LDFN model.

HSIs have four characteristics: band correlation, high resolution, mass of data and spectral variability. In order to solve these problems, the proposed deep CNN consists of local convolution and hybrid dilated convolution, which can not only extract rich spectral content but also rich spatial information. The framework of the proposed LDFN model is shown in Figure 3.

In Figure 3, the data of HSIs are pretreated. In the method of preprocessing, which is named principal component analysis (PCA) [28], an algorithm removes some useless bands at first, then the HSI is processed by reducing the dimension. It is carried out to pick up the most effective components of hyperspectral information and then patch blocks centered on label pixels are extracted to train LDFN. The overall process of LDFN is as follows: The original input size of the image block is set to $X\in R^{\left(H,W,C\right)}$, where H, W represent the height and width of the image in the space dimension, and *C* represents the number of bands in the spectral dimension. First, input the image block into a 3×3 two-dimensional convolutional layer. After that, the main channel is divided into two parts. On the one hand, the image block passes upward through two 1×1 local convolutional layers. On the other hand, the image block descends into a hybrid dilated convolution block (HDC), which is composed of a stack of dilated convolution layers with dilation rates of 2, 3 and 5. Then, the features are integrated, which generates a composite layer and then are fed into a residual block. In the residual block, two 3×3 convolution layers are used to extract input features and generate an output features layer, then the cross-layer connection is proposed to concatenate the HDC layer, the composite layer and the output features layer.

Then, the fused feature map passes through a 1 × 1 two-dimensional convolutional layer, a 2 × 2 average pooling layer and a global average pooling layer. Finally, the high-level features are input to the softmax layer to predict the classification label.

The number of filters is 48 except first the convolution’s filters are 16, batch normalization and relu activation are required after each convolution operation except the first local convolution. The size of the dilation rate in HDC is 2, 3 and 5. Last but not least, dropout is required in two local convolutions, the first one is 0.2, the other is 0.5.

## 3. Results

### 3.1. Datasets and Baseline

**Figure 4:**
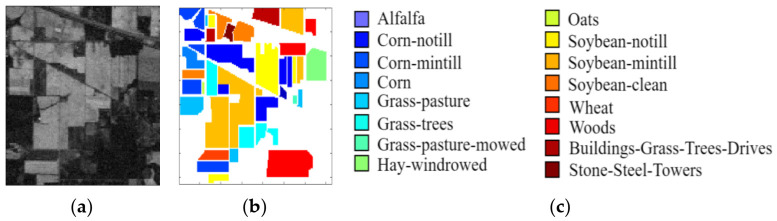
Indian Pines image. (**a**) Sample band of Indian Pines dataset. (**b**) Ground truth data. (**c**) Color band.

**Figure 5:**
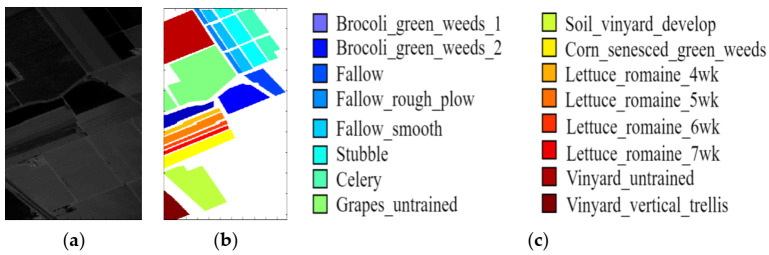
Salinas image. (**a**) Sample band of Salinas dataset. (**b**) Ground truth data. (**c**) Color band.

**Figure 6:**
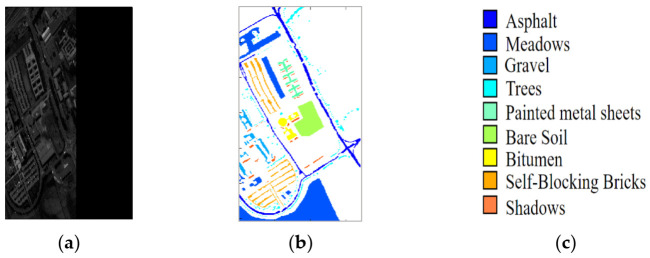
University of Pavia image. (**a**) Sample band of Pavia University dataset. (**b**) Ground truth data. (**c**) Color band.

In this paper, three benchmark hyperspectral datasets were used to verify the effectiveness of the proposed LDFN model, which includes the three datasets of the Indian Pines, the Salinas and the University of Pavia. Figure 4 shows the image of the band, the ground truth and color code of the Indian Pines dataset, Figure 5 shows the band image, the ground truth and color code of the Salinas dataset and Figure 6 shows band image, the ground truth and color code of the University of Pavia dataset.

Supervised learning needs a lot of label data, but hyperspectral label data is rare and the labeling process is very complex. Therefore, the experiment uses small samples, which are able to availably resolve the problem of the insufficient labels of hyperspectral data samples. The proportion of training samples in the three datasets is less than 10%. 

The Indian Pines dataset consists of 145×145 pixels and 224 spectral reflectors, the wavelength range is 0.4–2.5 μm with a spatial resolution of 20 m. The segmentation details of samples are listed in Table 1.

The Salinas dataset was collected over Salinas Valley in California. The area covered by 217 samples and Salinas ground truth also contains 16 classes. The segmentation details of the samples in the Salinas dataset are listed in Table 2.

The dataset from Pavia University was collected during a flight over Pavia in northern Italy. The size of image pixels is 610×610 and the geometric resolution is 1.3 m. The segmentation details of samples in the University of Pavia dataset are listed in Table 3.

The proposed LDFN model is established on tensorflow2.0 and the keras framework, it uses the programming language python. The experiments are trained and tested on a Geforce GTX 1660 GPU, RAM 16.00 GB. The Adam optimizer [29] is adopted and the epochs are 100 with the mini-batch size of 64. The training group and the test group are divided according to the ratio of 1:9. The initial learning rate is 0.001. To unify the input pixels, in the three used datasets, a pair of adjacent pixel units with the same size is fed into the model.

**Figure 7:**
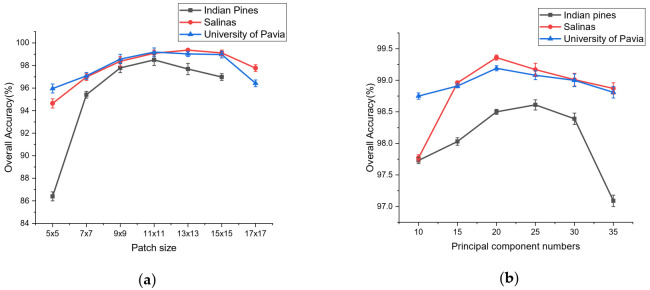
Overall accuracy (%) with different hyperparameters on three datasets. (**a**) patch sizes, (**b**) principal component numbers.

Figure 7 shows the accuracy of OA obtained by LDFN for the three used datasets with different hyperparameters.

1. According to the analysis of the curve in Figure 7a, first, the principal component numbers in the three used datasets are set to 20, then the tendency of curves can be seen clearly in the picture that OA increases rapidly first and gradually goes into a steady state, then curves drop which indicates that larger or smaller patch blocks cannot make the model stable and optimal. Therefore, the size of the patch is set to 11×11, 13×13 and 11×11 for Indian Pines, Salinas and University of Pavia, respectively.
2. Figure 7b reveals the curve changes with principal component numbers. First, the patch size in the three datasets is set to 11×11. As can be seen, the curves of overall accuracy increase till a steady state then drop down, which means that a reasonable expansion of the principal component numbers is conducive to the extraction of rich spectral information, but if the principal component numbers are excessive, it can lead to a decline in the performance of the network. Therefore, the principal component numbers are set to 25, 20 and 20 for the Indian Pines, Salinas and the University of Pavia, respectively.

### 3.2. Quantitative Metrics and Compared Methods

Three evaluation indexes are used in HSI classification to evaluate the model performance of different methods. Three objective metrics are used, that is, overall accuracy (OA), average accuracy (AA) and the Kappa coefficient. 

The proposed LDFN is in contrast with different other methods. The comparison methods can be generally split into two groups. One is the traditional machine learning method, including SVM[5]. The other consists of deep learning methods, including 3D-CNN[19], 3D-CAE[20], D-CNN[22] and SSRN[21]. The different methods have the same input size of patch blocks as our LDFN model.

### 3.3. Classification Results

**Figure 8:**
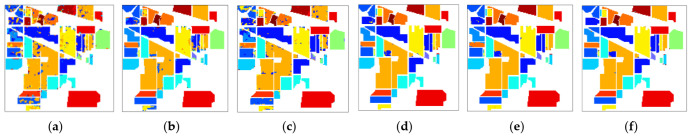
Classification maps for the Indian Pines dataset. (**a**) SVM:80.01%. (**b**) 3D-CNN:94.10%. (**c**) 3D-CAE:92.04%. (**d**) D-CNN:97.93%. (**e**) SSRN:98.09%. (**f**) LDFN:98.54%.

The first one is carried out on the dataset of the Indian Pines. All methods choose 10% samples to train the model and 90% samples to test. Table 4 reveals the quantitative results of the different methods and specific results are under the average of 10 training results. It is obvious that the accuracies of SVM, 3D-CNN and 3D-CAE are less than 95% in the three metrics above. D-CNN with contextual deep CNN framework and SSRN with several residual blocks have more than 95% accuracy in the three metrics. Overall, our proposed LDFN model has a better performance than the other methods in the three metrics. Figure 8 reveals the classification maps of the different methods clearly for the Indian Pines dataset. SVM has serious noise, 3D-CNN and 3D-CAE are smoother than SVM but still have some obvious noise in vision. SSRN and LDFN perform well and have less noise, furthermore, our LDFN model is better than SSRN in terms of detail.

**Figure 9:**
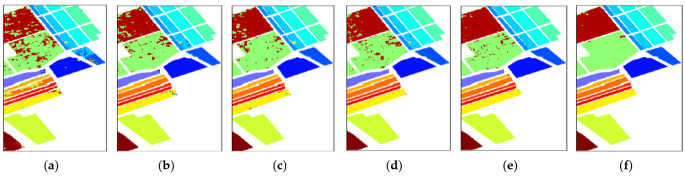
Classification maps for the Salinas dataset. (**a**) SVM: 85.97% (**b**) 3D-CNN: 95.24% (**c**) 3D-CAE: 96.05% (**d**) D-CNN: 95.35%. (**e**) SSRN: 98.38%. (**f**) LDFN: 99.36%.

The second one is based on the dataset of Salinas. A 1% sample was chosen to train the model and 99% to test all the methods. Table 5 reveals the quantitative results of different methods and specific results are under the average of 10 training results. It is obvious that the traditional method of SVM only has a classification accuracy of around 80%. However, the methods of deep learning basically have more than 95% accuracy. The deepening of the network layers in 3D-CNN, 3D-CAE and D-CNN can reach up to an accuracy of 95%. Our OA is 99.36%, AA is 99.56% and Kappa coefficient is 98.29%. Figure 9 reveals the classification diagrams of the Salinas dataset, which clearly shows that the LDFN model is smoother than other compared methods. Therefore, the performance of our LDFN model is better.

**Figure 10:**
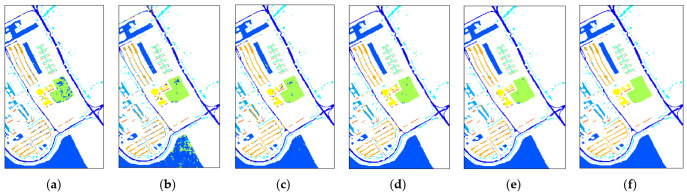
Classification maps for the University of Pavia dataset. (**a**) SVM: 89.18% (**b**) 3D-CNN: 94.33% (**c**) 3D-CAE: 95.36% (**d**) D-CNN: 97.19% (**e**) SSRN: 98.57%. (**f**) LDFN: 99.19%.

The third experiment is carried out on the dataset of Pavia University for which 2% of the samples are selected to train all methods’ models and 98% to test all the methods. Table 6 reveals the quantitative results of different methods and the specific results are under the average of 10 training results. As it can be seen, the OA of our LDFN model is 99.19%, AA is 98.89% and Kappa is 98.92%, which shows better performance than the 98.57% OA, 97.16% AA and 98.27% Kappa in SSRN as well as some other deep learning methods that have accuracies around 95%. Due to the loss of use in spatial features, the traditional method of SVM can only reach up to an accuracy of 80% on average. Figure 10 shows the classification maps of different methods for the University of Pavia dataset, which intuitively presents that our LDFN model has better performance in terms of vision, especially for the details of the edge and local parts.

### 3.4. Comparison of Different Local and HDC Fusion Strategies

In this section, different structures of local and HDC fusion models are compared to prove the effectiveness of the LDFN model. Since the local spatial-spectral contents are extracted by local convolution, the size of the HDC starts at 2 instead of 1. Table 7 reveals the values of OA obtained from the local and HDC fusion models. The LDFN24 represents the dilation rates stacked by size 2 and 4, LDFN25 is stacked by size 2 and 5, LDFN34 consists of size 3 and 4. Particularly, LDFN234 consists of three different dilation rates, the fusion dilation size is 2, 3 and 4. It is obvious in Table 7 that the model with the local and HDC structure achieves better HSI classification results than the compared fusion model D-CNN. Meanwhile, according to accurate experiment results, the proposed LDFN indeed performs better than other methods with different sizes of dilation rates.

In general, the extensive experiments with three HSI datasets prove that our LDFN model is not only steady and convenient for training, but also effective and advanced in technology.

## 4. Conclusions

In this paper, a novel deep learning method called the local and hybrid dilated convolution fusion network was proposed for HSI classification. The proposed local and hybrid dilation fusion network fuses local convolution and hybrid dilation convolution, local convolution connects local pixels closely, which can make our convolution layer more flexible and expressive. Hybrid dilation convolution stacked with different dilation rates of 2, 3 and 5 can raise the field of vision and consider the spatial correlation of hyperspectral images in adjacent areas without increasing the amount of computation. It also uses the specific residual fusion network to integrate the previous HDC and standard convolution into the main channels, which can not only solve the problem of the insufficient receptive field but also can extract multi-scale feature information. Experimental results demonstrate that the LDFN model can achieve a satisfactory classification accuracy for hyperspectral images under the lightweight standard.

The proposed LDFN model still has great room for improvement. At present, the LDFN model still has redundant parameters and needs to spend some time training to extract spectral-spatial features. In future research, more attention will be paid to multi-scale information fusion and reducing model parameters, which may help optimize the LDFN model and can better integrate spectral features and spatial features for HSI classification.

## Figures and Tables

**Table 1:** The Number of Samples for the Indian Pines Dataset.

#	Class	Samples	Train	Test
1	Alfalfa	46	5	41
2	Corn-notill	1428	143	1285
3	Corn-mintill	830	83	747
4	Corn	237	24	213
5	Grass-pasture	483	48	435
6	Grass-trees	730	73	657
7	Grass-pasture-mowed	28	3	25
8	Hay-windrowed	478	48	430
9	Oats	20	2	18
10	Soybean-notill	972	97	875
11	Soybean-mintill	2455	245	2210
12	Soybean-clean	593	59	534
13	Wheat	205	20	185
14	Woods	1265	126	1139
15	Buildings-Grass-Trees-Drives	386	39	347
16	Stone-Steel-Towers	93	9	84
Total		10,249	1024	9225

**Table 2:** The Number of Samples for the Salinas Dataset.

#	Class	Samples	Train	Test
1	Brocoli_green_weeds_1	2009	20	1989
2	Brocoli_green_weeds_2	3726	37	3689
3	Fallow	1976	20	1956
4	Fallow_rough_plow	1394	14	1380
5	Fallow_smooth	2678	27	2651
6	Stubble	3959	39	3920
7	Celery	3579	36	3543
8	Grapes_untrained	11,271	113	11,158
9	Soil_vinyard_develop	6203	62	6141
10	Corn_senesced_green_weeds	3278	33	3245
11	Lettuce_romaine_4wk	1068	11	1057
12	Lettuce_romaine_5wk	1927	19	1908
13	Lettuce_romaine_6wk	916	9	907
14	Lettuce_romaine_7wk	1070	11	1059
15	Vinyard_untrained	7268	72	7196
16	Vinyard_vertical_trellis	1807	18	1789
Total		54,129	541	53,588

**Table 3:** The Number of Samples for the University of Pavia Dataset.

#	Class	Samples	Train	Test
1	Asphalt	6631	132	6499
2	Meadows	18,649	373	18,276
3	Gravel	2099	42	2057
4	Trees	3064	61	3003
5	Painted metal sheets	1345	27	1318
6	Bare Soil	5029	100	4929
7	Bitumen	1330	27	1303
8	Self-Blocking Bricks	3682	74	3608
9	Shadows	947	19	928
Total		42,776	855	41,921

**Table 4:** Classification Results of Different Methods for the Indian Pines Dataset.

Class	SVM[5]	3D-CNN[19]	3D-CAE[20]	D-CNN[22]	SSRN[21]	LDFN
1	67.05	98.00	90.48	95.24	97.82	100.00
2	93.77	96.12	92.49	97.66	99.16	99.50
3	67.55	80.49	90.37	97.72	97.11	96.02
4	61.20	92.00	86.90	97.70	97.51	99.05
5	93.15	97.00	94.25	97.63	99.24	99.54
6	95.70	96.77	97.07	99.16	98.57	99.09
7	84.00	98.02	91.26	97.20	98.70	100.00
8	90.52	98.35	97.79	99.08	99.70	100.00
9	75.05	86.30	75.90	93.33	98.53	100.00
10	67.70	90.65	87.34	97.16	98.27	97.27
11	87.61	90.17	90.24	95.53	97.18	96.90
12	61.21	92.60	95.76	96.17	97.12	97.47
13	92.01	97.00	97.49	98.53	99.00	100.00
14	88.77	97.85	96.03	98.37	99.17	99.22
15	88.81	96.43	90.48	97.06	99.20	99.12
16	90.71	97.00	98.82	93.23	97.82	97.62
OA	80.01	94.10	92.04	97.93	98.09	98.54
AA	81.55	94.05	92.35	96.92	98.38	98.80
Kappa	78.33	93.48	92.21	95.17	97.01	98.34

**Table 5:** Classification Results of Different Methods for the Salinas Dataset.

Class	SVM[5]	3D-CNN[19]	3D-CAE[20]	D-CNN[22]	SSRN[21]	LDFN
1	80.00	97.54	99.00	97.20	99.23	100.00
2	87.94	98.89	98.29	96.92	99.94	100.00
3	89.72	97.42	96.13	83.62	99.95	100.00
4	82.55	98.10	97.34	96.28	97.49	98.22
5	77.87	97.98	97.35	94.76	96.70	100.00
6	88.67	97.97	97.90	95.07	99.15	99.90
7	89.86	98.71	97.64	97.12	99.62	100.00
8	81.33	89.67	91.58	90.84	98.16	98.53
9	90.02	98.99	98.93	97.07	99.96	99.55
10	86.57	96.27	95.98	96.43	99.43	99.81
11	90.00	98.48	98.37	95.87	97.16	100.00
12	84.06	98.76	98.84	95.64	98.53	99.95
13	58.19	95.88	98.56	96.24	95.81	99.66
14	57.49	98.94	97.52	95.10	98.53	98.69
15	69.81	86.18	88.85	96.03	99.08	98.69
16	89.56	98.70	97.34	95.11	99.35	100.00
OA	85.97	95.24	96.05	95.35	98.38	99.36
AA	81.48	96.78	96.85	94.96	98.63	99.56
Kappa	83.93	94.66	95.51	95.46	98.36	99.29

**Table 6:** Classification Results of Different Methods for the University of Pavia Dataset.

Class	SVM[5]	3D-CNN[19]	3D-CAE[20]	D-CNN[22]	SSRN[21]	LDFN
1	90.36	93.27	95.21	96.11	98.80	99.17
2	97.25	97.61	96.06	98.91	99.69	99.95
3	70.93	90.01	91.32	90.82	95.15	94.64
4	90.93	94.17	98.28	92.63	95.02	99.53
5	96.46	98.02	95.55	97.63	99.14	100.00
6	81.76	90.03	95.30	99.14	99.69	99.92
7	83.59	80.21	95.14	93.12	96.68	99.85
8	88.14	95.97	91.38	97.77	98.74	97.24
9	96.97	99.63	99.96	89.43	91.54	99.78
OA	89.18	94.33	95.36	97.19	98.57	99.19
AA	88.48	93.21	95.35	95.06	97.16	98.89
Kappa	88.63	93.07	95.12	96.29	98.27	98.92

**Table 7:** OA Values Obtained by Local and HDC Fusion Model on Three Datasets.

Dataset	Metric	D-CNN	LDFN24	LDFN25	LDFN34	LDFN234	LDFN
Indian Pines	OA	97.93	98.09	98.01	97.92	98.25	98.54
Salinas	OA	95.35	99.01	98.47	98.12	99.11	99.36
University of Pavia	OA	97.19	98.59	98.30	97.79	99.07	99.19

## Data Availability

Some or all data used during the study are available online in accordance with funder data retention policies. (http://www.ehu.eus/ccwintco/index.phptitle=Hyperspectral_Remote_Sensing_Scenes, accessed on 22 February 2021).
